# Health Information System

**DOI:** 10.1590/S1679-45082013000400001

**Published:** 2013

**Authors:** Valéria Pinheiro de Souza, Oscar Fernando Pavão dos Santos, Nelson Wolosker

**Affiliations:** 1 Sociedade Beneficente Israelita Brasileira Albert Einstein, São Paulo, SP, Brazil; 2 Hospital Israelita Albert Einstein, São Paulo, SP, Brazil

More than a hundred years after its foundation, the Institute of Medicine (IOM) published a report called To err is human: building a safer health system, stating that between 44 thousand and 98 thousand patients die every year in the United States due to potentially preventable medical errors^(1)^. With the intention of improving quality of healthcare and, as a consequence, decreasing the rates of adverse events, this report guided the implementation of electronic records and other forms of technology in Medicine^(2)^, defining the goal of enhanced safety, effectiveness, quick access, efficiency, and equity^(2)^.

Two questions must be asked when considering this strategy:

1. Would the implementation of the electronic record be a guarantee of achieving the quality goals determined by the IOM?
2. Can one have return on investment with the implementation of the electronic record?

In regard to the first question, while the benefits of information technology are theoretically clear in almost every area of society, applying them to healthcare has been difficult, and also their use has been limited^(2)^. Another aspect is that most applications focus in financial and administrative information, more than in information on care^(2)^.

Many studies have demonstrated gains in clinical practice by adopting the electronic record. Chaudhry et al.^(3)^ conducted a study with four organizations (Regenstrief Institute, Brigham and Women's Hospital/Partners HealthCare, US Department of Veterans Affairs and LDS Hospital/Intermountain Health Care) and identified five opportunities of improvement: better compliance to protocols; capacity to increase survival, chronic disease monitoring and their care; reducing the rate of medication errors; and finally, reducing the use of unnecessary resources^(3)^. As an example, the Brigham and Women's Hospital, in Boston, demonstrated a reduction in the rate of errors from 10.7 to 4.9 per 1000 patients-day (drop by 55%) with the use of electronic prescriptions^(3)^.

The factors that should be considered when an information system is implemented in healthcare include choice of software; infrastructure costs; staff knowledge on technology; parallel projects that may compete with the implementation; goals of leadership; project governance, and system development^(4)^.

Four variables are considered as most important for the success of implementation and maintenance of the systems:

1. end user understanding of the long-term benefits and impact in daily practice;
2. organization infrastructure to support implementation;
3. language patterns; organizational and national rules;
4. define objective and metrics for each phase of implementation^(4)^.

Of these four variables, the most important and greatest challenge, in our view, is understanding the benefits and impact on daily practice by the end user, since this activity definitely demands time using the computer in care tasks. It is important users understand that, after the initial phase - the learning curve -, the time spent per patient becomes acceptable and the benefits worthy, such as having information in real time and centralized in the patient, besides the support to quick and safe decision making^(4)^.

In regard to return on investment in electronic chart implementing, discussions on Information Technology productivity are new in healthcare. In other sectors of economy, though, productivity is highly used. In other industries, the use of productivity as metrics for the service industry presents flaws, considering that conceptually productivity is the ratio between what is produced and the resources necessary to produce it^(5)^. A good example of this situation is physicians who communicate with their patients by means of telephone messages (SMS), e-mails or telephone calls. Replacing the office visits may seem less productive if the number of visits is considered as metrics, but if we consider accessibility and care management, those physicians may be more effective, providing better care to the patients^(5)^.

Information technology use has already been defined by the IOM as the means to obtain quality in healthcare.

The use of information technology for prevention of adverse events is more effective when compared to overseeing and reverting the consequences of an error.

Without information systems implemented and sufficiently integrated, which support decisions in real time and increase safety during care, organizational leaders will be nothing but well-intentioned activists.

As for the return on investment, we must create new metrics for productivity in health, focusing on strategies that increase usability and control over-optimistic expectations about financial return.
